# Surface-layer protein is a public-good matrix exopolymer for microbial community organisation in environmental anammox biofilms

**DOI:** 10.1038/s41396-023-01388-y

**Published:** 2023-03-04

**Authors:** Lan Li Wong, Yang Lu, James Chin Shing Ho, Sudarsan Mugunthan, Yingyu Law, Patricia Conway, Staffan Kjelleberg, Thomas Seviour

**Affiliations:** 1 Singapore Centre for Environmental Life Sciences Engineering, Nanyang Technological University, Singapore, 637551 Singapore; 2 School of Biological Sciences, Nanyang Technological University, Singapore, 637551 Singapore; 3 The Australian Centre for Ecogenomics, School of Chemistry and Molecular Biosciences, The University of Queensland, St Lucia, QLD 4072 Australia; 4 School of Biological, Earth and Environmental Sciences, University of New South Wales Sydney, Sydney, 2052 Australia; 5 WATEC Aarhus University Centre for Water Technology, Universitetsbyen 36, Bldg 1783, 8000 Aarhus, Denmark

**Keywords:** Biofilms, Microbial communities, Community ecology

## Abstract

Extracellular polymeric substances (EPS) are core biofilm components, yet how they mediate interactions within and contribute to the structuring of biofilms is largely unknown, particularly for non-culturable microbial communities that predominate in environmental habitats. To address this knowledge gap, we explored the role of EPS in an anaerobic ammonium oxidation (anammox) biofilm. An extracellular glycoprotein, BROSI_A1236, from an anammox bacterium, formed envelopes around the anammox cells, supporting its identification as a surface (S-) layer protein. However, the S-layer protein also appeared at the edge of the biofilm, in close proximity to the polysaccharide-coated filamentous *Chloroflexi* bacteria but distal to the anammox bacterial cells. The *Chloroflexi* bacteria assembled into a cross-linked network at the edge of the granules and surrounding anammox cell clusters, with the S-layer protein occupying the space around the *Chloroflexi*. The anammox S-layer protein was also abundant at junctions between *Chloroflexi* cells. Thus, the S-layer protein is likely transported through the matrix as an EPS and also acts as an adhesive to facilitate the assembly of filamentous *Chloroflexi* into a three-dimensional biofilm lattice. The spatial distribution of the S-layer protein within the mixed species biofilm suggests that it is a “public-good” EPS, which facilitates the assembly of other bacteria into a framework for the benefit of the biofilm community, and enables key syntrophic relationships, including anammox.

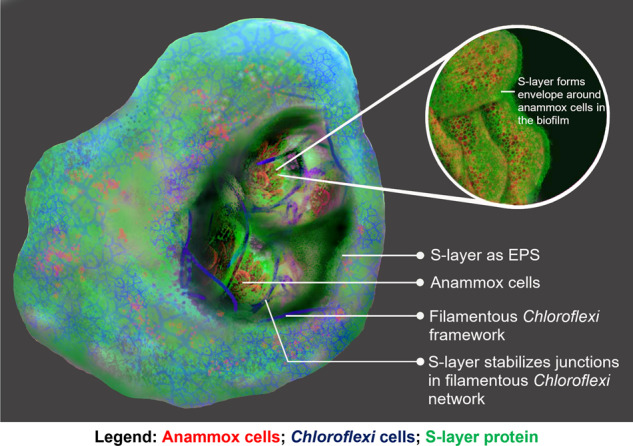

## Introduction

Extracellular polymeric substances (EPS), including polysaccharides, proteins and nucleic acids, aid the adhesion and aggregation of bacteria, and form extracellular scaffolds that promote biofilm formation [1–3]. Biofilms have unique three-dimensional structures, with the scaffold providing stratification of physicochemical properties, and enabling different ecological niches to form in response to, for example, oxygen and nutrient availability [4]. Microorganisms embedded within biofilm matrices display emergent properties, such as enhanced stress tolerance, and form synergistic relationships with other microbial populations [5, 6]. Such synergies and micro-domains contribute to microcolony development and allow microbial growth also under bulk conditions of nutrient limitation [1, 7]. Hence, there is a strong need to resolve molecular, structural and functional aspects of microbial EPS and their role in biofilm assembly and function [8]. For example, biofilm community spatial organisation is central to industrial biotechnologies, such as activated sludge in wastewater purification, and the increased virulence of pathogenic biofilms, as exemplified by periodontitis [9–11]. Despite their importance for community biofilms, most studies into the roles of EPS in biofilm architecture and function have focused on laboratory cultures of single or defined mixed species rather than naturally occurring biofilms [6, 10, 12–16], which may not realistically represent bioprocesses occurring in environmental or industrial systems.

The biofilm-based multispecies anaerobic ammonium oxidation (anammox) process accounts for up to 80% of oceanic nitrogen losses [17]. It is also employed extensively in industry as a side stream in wastewater treatment processes, where it outperforms conventional nitrogen removal by allowing direct transformation of ammonium to nitrogen using nitrite as an electron acceptor, with minimum requirement for organic substrates [18]. Members of the *Planctomycetes* perform the anammox process in such communities, with members of *Chlorobi, Bacteroidetes, Chloroflexi* or *Proteobacteria* commonly present as co-occurring species [19, 20]. A symbiotic relationship has been described for anammox bacteria and *Chloroflexi*, which utilise organic matter secreted by anammox bacteria. While *Chloroflexi* has been suggested to form filamentous scaffolding to facilitate biofilm formation, this is yet to be demonstrated for anammox biofilms [21, 22].

Regardless of habitat, whether natural or engineered, anammox bacteria always express EPS and form biofilms, either floccular, granular or surface-attached, that enable syntrophic relationships with other microorganisms [23]. Residing as microbial consortia enhances nitrogen removal efficiency, increases tolerance to stress, and, crucially for wastewater treatment, improves biomass retention and hydraulic throughput [21, 24]. Hence, anammox biofilms are ideal model systems for understanding the role of EPS in microbial communities. Anammox EPS have accordingly been studied extensively, with the classes of biopolymers present and their impact on biofilm biophysical and mechanical properties, such as attachment, self-aggregation and settleability, being described [25–27]. β-sheet proteins, which have been identified as putative surface (S-) layer proteins, are consistently detected in the EPS of anammox biofilms [9, 28]. While S-layer proteins are present extracellularly as a paracrystalline lattice on the outer side of the cell envelope layer of microorganisms, they are not generally recognised as biofilm matrix components per se (i.e., beyond contributing to surface attachment) [29]. However, S-layer protein expression has been shown to correlate with biofilm formation in several species, including *Clostridium difficile* and *Tannerella forsythia* [30, 31].

In this study, we resolve the contribution of an EPS to polymicrobial community organisation within an anammox biofilm, and describe the structural and functional roles of the S-layer protein in the extracellular matrix. We analysed the spatial distribution of anammox extracellular protein, BROSI_A1236, within the biofilm, specifically relative to *Candidatus* Brocadia sinica and *Chloroflexi* using an antibody raised against it. These bacteria generally predominate in synthetic wastewater-enriched anammox granular sludge [19, 21, 32, 33]. We could thus infer that, in addition to its role as an S-layer protein, the extracellular protein accumulates in the matrix at the biofilm edges as well as at the borders of anammox cell clusters and fortifies junctions in the *Chloroflexi* network. The S-layer protein-*Chloroflexi* association builds an EPS-mediated scaffold for the anammox bacteria [22, 34]. These findings inform on the spatial organisation of a key environmental and engineered community biofilm, as mediated by its biopolymers in the biofilm matrix.

## Materials and methods

### Anammox granules cryosection

Anammox granules collected freshly from a bioreactor [35] were washed twice with 1x phosphate buffer saline (PBS, 137 mM NaCl, 2.7 mM KCl, 10 mM Na_2_HPO_4_, and 1.8 mM KH_2_PO_4_, pH7.4) followed by fixation with 4% paraformaldehyde (PFA) overnight. The granules were then washed with 1x PBS and placed in 15% sucrose followed by 3:1, 1:1 and 1:3 15% sucrose: OCT (v/v) and finally 100% OCT sequentially overnight. OCT-treated anammox granules were transferred to a square mould and stored at −20 °C overnight. The sample was sliced on Leica CM 1950 cryostat instrument to obtain 2–10 µm thin slices of anammox biofilm on a poly-L-lysine coated slide (Sigma-Aldrich). The slide was then dehydrated for 3 min sequentially in 50%, 70%, and 98% (v/v) ethanol solutions. The slides were air-dried and kept at room temperature for fluorescent labelling.

### Fluorescence microscopy

Microscopic imaging was conducted on a Zeiss LSM 780 confocal microscope with a 63x/1.4 oil objective. Briefly, cryosectioned anammox granules were stained with Concanavalin A, Alexa Fluor 594 conjugate (0.2 mg/mL (w/v)), SYTO 9 from BacLight Live/Dead viability stain and SYPRO Ruby stain according to manufacturers’ manuals. All dyes were obtained from Thermo Fischer Scientific.

### Microbial community profiling and data analysis

Microbial community profiling of the reactors was conducted weekly [36]. The average abundance of the microbial community at the family level from reactors over two months (k = 6) was used to construct a Krona plot [37]. Metagenomic analysis was carried out on DNA extracted from granules according to a previously described method [38]. Metagenome assembled genomes (MAGs) associated to *Chloroflexi* were selected and inserted into a species tree [39] in Kbase [40]. The quality of the MAGs was assessed using CheckM for completeness, contamination and strain heterogeneity (SI Table 1) [41].

### Protein sequence aligner

Translated protein sequences of BROSI_A1236 were obtained from NCBI (Locus: GAN32721, accession BAFN01000001), and utilised as database for diamond blastx search on *Chloroflexi* MAGs 1–5, with default settings [42, 43].

### Scanning electron microscopy (SEM) and variable pressure SEM (VP-SEM)

Freeze-dried fresh anammox granules were sputtered with 6 nm platinum using Leica ACE200 Sputter coater (Leica Microsystems, Wetzlar, Germany). SEM imaging was performed with Hitachi FlexSEM 1000 II (Hitachi, Tokyo, Japan) at 5 kV at high vacuum image mode using SE detector. For samples without platinum coating, variable pressure SEM (VP-SEM) imaging was performed at 10 kV at 30 Pa using BSE detector.

### Antibody generation

Two rabbits were inoculated with purified BROSI_A1236 isolate [35] in a three-month immunisation protocol consisting of three injections on days 0, 7, and 14 (iDNA Biotechnology Pte Ltd). The serum was collected from rabbit antiserum. Crude serum of one rabbit was used as anti-BROSI_A1236 primary antibody.

Binding specificity of the antibody was validated by western blot, a primary antibody concentration-dependent decrease in fluorescence signal, as well as the absence of the fluorescence signal when the primary antibody was omitted (SI Fig. 1).

### Immunofluorescence-fluorescence in situ hybridisation (FISH) staining

A slide with cryosectioned anammox biofilm, as described above, was blocked overnight with PBS-T (137 mM NaCl, 12 mM PO_4_^3-^, 2.7 mM KCl, 0.05% Tween 20, pH 7.4) and 5% (w/v) bovine serum albumin (BSA) at 4 °C. The primary antibody was diluted 200 or 250 times in blocking buffer with RNase inhibitor (0.4 U/µL, Thermo Fisher Scientific) and incubated for 1.5 h at 22 °C. The slide was then washed three times with PBS-T for 5 min followed by incubation with 500 times diluted goat anti-rabbit IgG (H + L)-AF488 or AF405 secondary antibody (Thermo Fisher Scientific) in blocking buffer for 1 h at 22 °C in the dark. The slide was washed three times for 5 min with PBS-T followed by fixing with 4% PFA for 30 min. Next, the fixed slide was washed two times with 1x PBS followed by sequential dehydration using 50%, 70% and 98% (v/v) ethanol solutions for 3 min. FISH was then performed on a dried slide with the probes listed in SI Table 2 [44]. The slide was then viewed using Zeiss LSM 780 confocal laser scanning microscopy. The biovolume of SYPRO Ruby and BROSI_A1236 signal on four biofilms cross-section was quantified using Imaris software.

### Image processing using ImageJ

Fluorescence intensity line profiles were obtained by drawing a line across the region of interest with a line pixel width of 18. The colocalisation profile of any two fluorescent channels of interest from the same image acquired was processed by applying Coloc 2 to perform the pixel intensity correlation over the space methods of Pearson. Pearson’s R-value without threshold was used in the data analysis and comparison.

### Sodium dodecyl sulfate-polyacrylamide gel electrophoresis analysis of the soluble EPS

Twenty microliters of the crude ionic liquid anammox biofilm extract was denatured in NuPAGE LDS sample buffer (Thermo Fischer Scientific) (1:1 (v/v)) for 10 min at 70 °C and were loaded onto a 12% (w/v) hand-cast polyacrylamide gel, according to the protocol provided by Bio-Rad. The electrophoresis was carried out at 165 V in tris-glycine running buffer (25 mM Tris, 0.192 M glycine, 0.1% (w/v) SDS, pH 8.3) for 60 min. Lane L was loaded with PageRuler Plus Prestained Protein Ladder, 10 to 250 kDa (Thermo Fischer Scientific, 22619).

### Immunoblot analysis

After electrophoresis, proteins were transferred from the SDS-PAGE gel to a membrane using an iBlot transfer system (Invitrogen). The polyvinylidene difluoride (PVDF) membrane was blocked with PBS-T and 5% (w/v) bovine serum albumin (BSA) and kept overnight at 4 °C. The primary antibody was then diluted 6000 times in blocking buffer and incubated for 2 h at 22 °C. The PVDF membrane was washed three times with PBS-T for 5 min before incubating with goat anti-rabbit IgG (H + L) secondary antibody, HRP (Thermo Fisher Scientific) diluted 10,000x in blocking buffer for 1 h at 22 °C in the dark. After incubation, the membrane was washed five times for 5 min with PBS-T. For immune detection, the blot was developed in 1:1 (v/v) dilution of SuperSignal West Femto Trial Kit (Thermo Fisher Scientific) to achieve the desired signal intensity. The blot was imaged using Amersham ImageQuant 800 Fluor Biomolecular Imager (cytiva).

## Results

### Extracellular biopolymers localise in distinct regions of *Ca*. B. sinica-enriched anammox biofilms

**Fig. 1:**
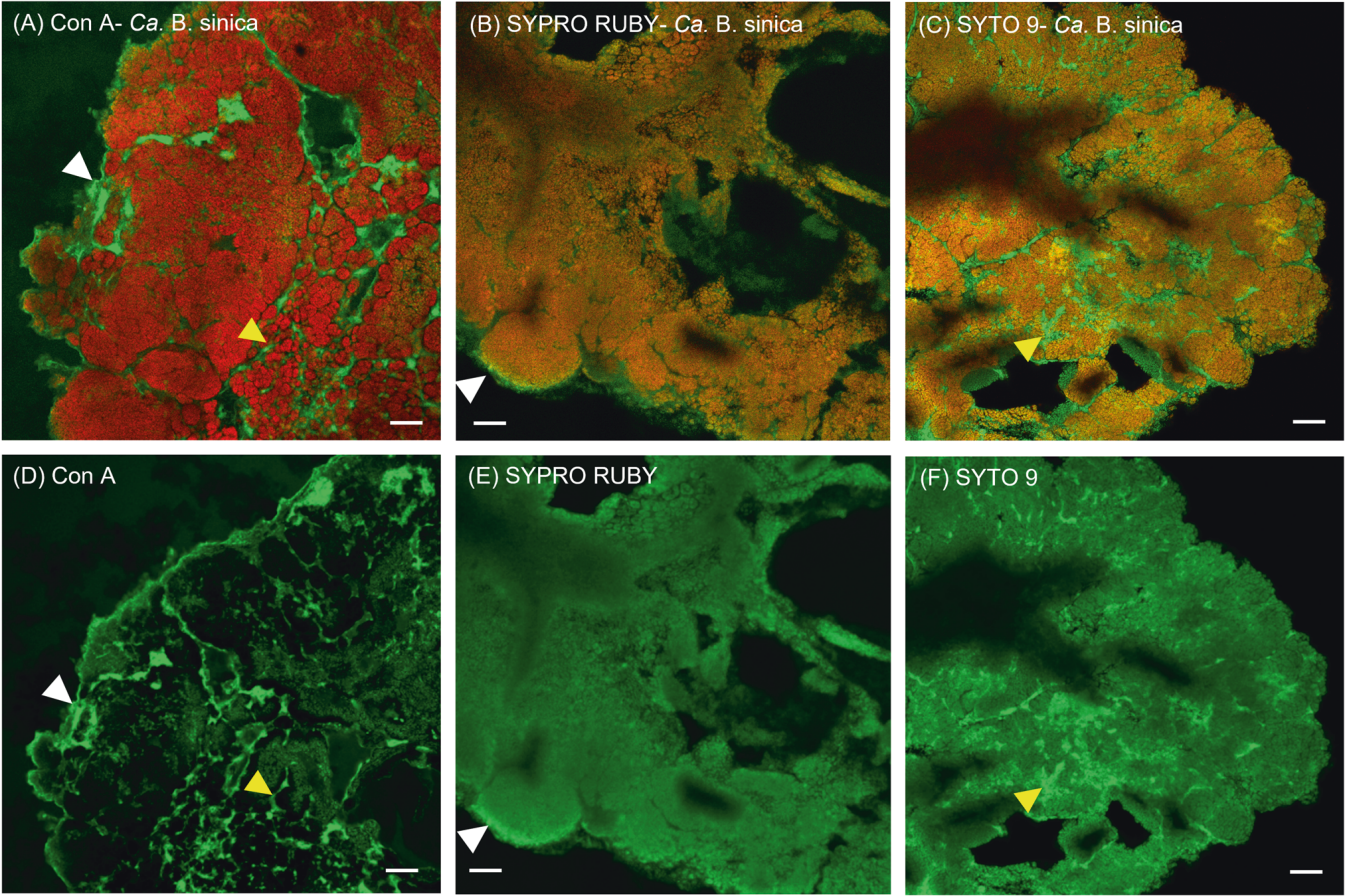
**A**–**C** Confocal laser scanning microscopy (CLSM) images of 5 μm anammox biofilm sections stained with *Ca*. **B**. sinica-specific FISH probe, Bsi630 (red) and EPS-specific dyes (green) targeting (**A**) polysaccharides (Con A), (**B**) proteins (SYPRO Ruby) and (**C**) nucleic acids (SYTO 9). Three compositionally distinct regions were identified where: (i) the periphery of the biofilm was enriched with polysaccharides and protein-specific stains (identified by white arrowheads); (ii) the internal *Ca*. B. sinica cell cluster borders were detected by the polysaccharides and nucleic acid-specific stains (indicated by yellow arrowheads); and (iii) SYPRO Ruby and SYTO 9 stained *Ca*. B. sinica bacterial cells, showing overlapping orange signals in 1B and 1C. **D**–**F** Single channel extracellular polymer stains of anammox biofilms. Scale bar indicates 20 μm.

Thin anammox biofilm sections were prepared and stained separately with three selected EPS-specific dyes in combination with a fluorescence in situ hybridisation (FISH) probe specific for the dominant population of *Ca*. B. sinica, Bsi630 (Fig. 1A–C) [45]. The three EPS dyes used, Concanavalin A (Con A), SYPRO Ruby and SYTO 9, are specific for polysaccharides, proteins and DNA, respectively. The staining patterns demonstrated three compositionally distinct regions within the biofilm section (Fig. 1). The first region encompassed the periphery of the biofilm, which was stained most intensely by Con A and SYPRO Ruby (Fig. 1A, D and Fig. 1B, E respectively with regions identified by white arrowheads). The second region consists of the internal channels between *Ca*. B. sinica cells, which stained positively to Con A and SYTO 9 (Fig. 1A, D and Fig. 1C, F with regions identified by yellow arrowheads). The final region displayed *Ca*. B. sinica-cell clusters which were stained by both SYPRO Ruby and SYTO 9 (Fig. 1B, C, E, F).

### *Ca*. B. sinica cell clusters are demarcated by a filamentous polysaccharide-rich *Chloroflexi*

**Fig. 2:**
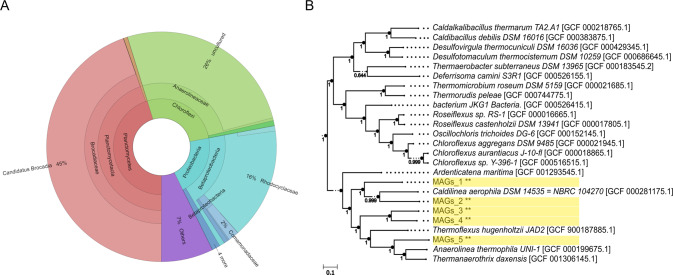
**A** Krona chart showing taxonomic classification of the laboratory enriched anammox granular biofilm based microbial communities at the phylum level according to 16S amplicon sequencing. **B** Phylogenetic tree generated using FastTree based on MAGs retrieved from anammox bioreactor metagenomic sequencing data. Only the closest identified sequences were selected. The scale indicates 0.1 nucleotide change per nucleotide position.

Metagenomic sequencing showed a high co-enrichment of *Ca*. B. sinica (45%) and *Chloroflexi* (26%) in the laboratory-enriched anammox granules (Fig. 2A). The phylogenetic tree of five metagenome assembled genomes (MAGs) recovered within the *Chloroflexi* phylum in the biofilm is presented in Fig. 2B. The coexistence of *Chloroflexi* with anammox bacteria in the biofilm is consistent with a previously described organic-free synthetic nutrient-fed bioreactor [21].

**Fig. 3:**
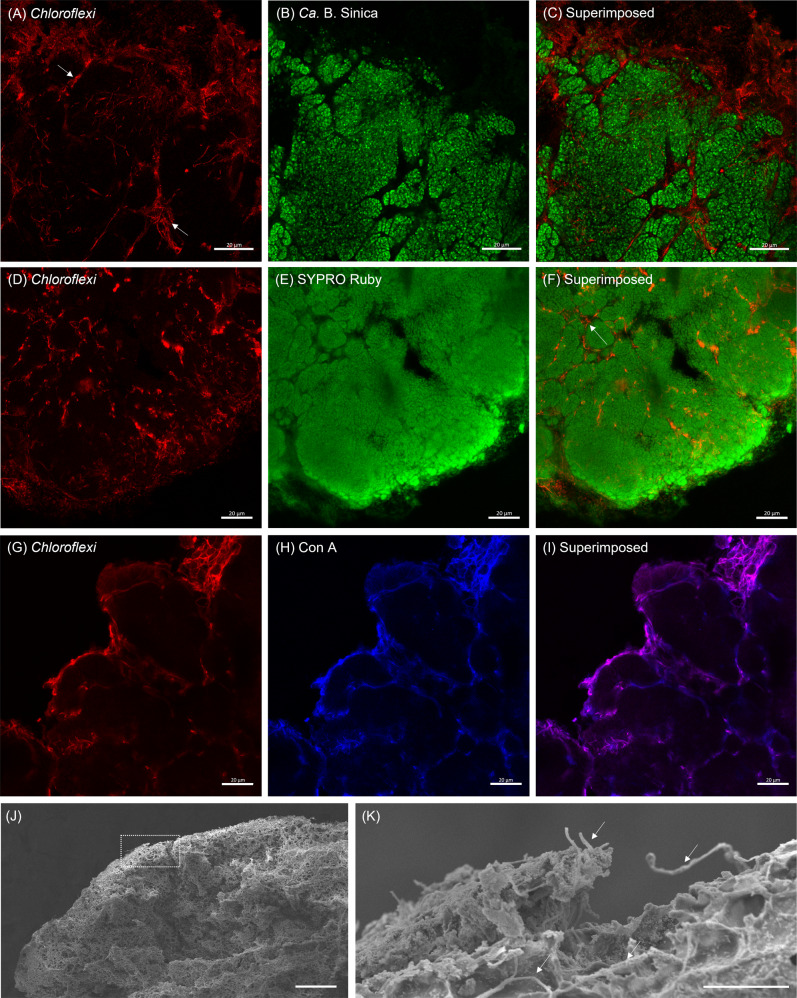
Confocal laser scanning microscopy (CLSM) images show 10 µm anammox biofilm sections stained with the general *Chloroflexi* phylum probes CFX1223 and GNSB941 (red) (**A**, **D,** and **G**). These are coupled separately with either (**B**) the *Ca*. B. sinica FISH probe, Bsi630 (green), (**E**) SYPRO Ruby (green) or (**H**) Concanavalin A (blue). **C**, **F**, **I** are the superimposed images of **A** and **B**, **D** and **E**, and **G** and **H**, respectively, where the white arrows denote the assembly of *Chloroflexi* filaments at the borders of anammox bacterial cell clusters. Scale bars indicate 20 μm. Scanning electron microscopy (SEM) images showing (**J**) a highly filamentous and networked structure at the surface of anammox biofilm and (**K**) an enlarged selected area of anammox biofilm edge in **J** that is likely made up predominately of filamentous *Chloroflexi* cells. Scale bars indicate 10 μm.

FISH microscopy using probes targeting the *Chloroflexi* phylum (i.e., CFX1223 and GNSB941), along with *Ca*. B. sinica, confirmed the presence of *Chloroflexi* in the anammox biofilms (Fig. 3A). The results demonstrated that *Ca*. B. sinica exists in tightly packed, mono-species clusters, and the *Chloroflexi* are located predominantly in the channels between and around *Ca*. B. sinica cell cluster borders as well as at the edge of the biofilm surface, where metabolically active *Ca*. B. sinica was absent (Fig. 3B, C, colocalisation Pearson’s R value, *r* = 0).

Localisation of *Chloroflexi* relative to anammox cells and EPS was assessed using combined extracellular polysaccharide and protein staining. The *Chloroflexi*, occupying the regions between *Ca*. B. sinica cell clusters, were only lightly stained, or not at all, by SYPRO Ruby (Fig. 3D–F, *r* = 0.26). In contrast, a strong Con A signal was observed only at the boundaries of anammox cell clusters and at the outer biofilm edge. The Con A signal overlapped almost entirely with the *Chloroflexi* FISH probe signal (Fig. 3G–I, *r* = 0.88).

Scanning electron microscopy (SEM) images of the freeze-dried anammox biofilm revealed the presence of a highly cross-linked network of filamentous bacteria at the edge of the biofilm (Fig. 3J, K and SI Fig. 2). The filamentous network at the biofilm edge is comprised of *Chloroflexi*, along with EPS matrix material (Fig. 3).

### Anammox S-layer protein coats *Ca*. B. sinica cells and occupies the space surrounding *Chloroflexi* cells at the edge of the biofilm

**Fig. 4:**
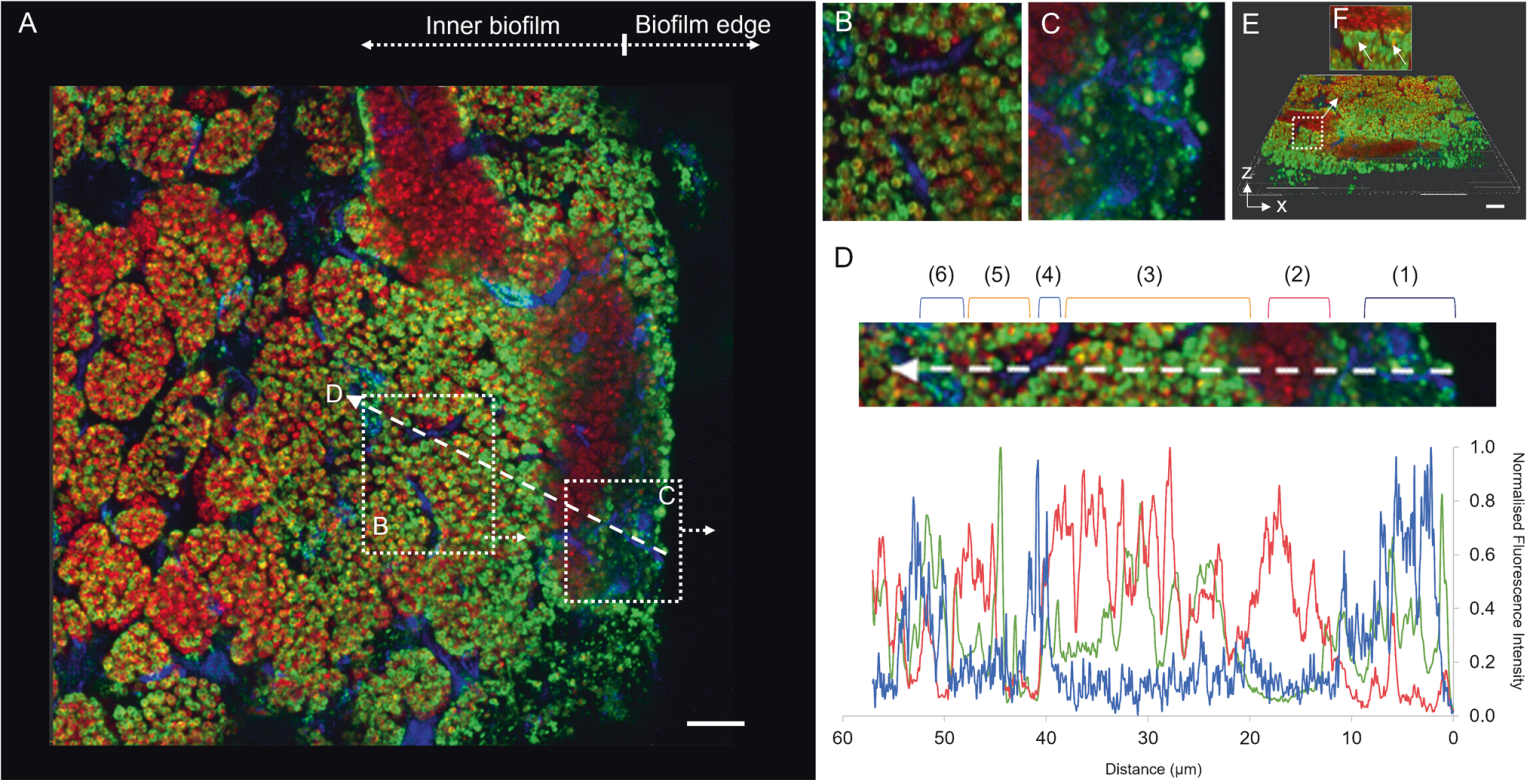
**A** Confocal laser scanning microscopy (CLSM) images of a thin anammox biofilm section (3 µm) stained with *Ca*. B. sinica FISH probe Bsi630 (red), S-layer protein antibody (250x dilution) visualised with Alexa Fluor 488-labelled goat anti-rabbit IgG (green) and general *Chloroflexi*-phylum probes CFX1223 and GNSB941 (blue). The *Ca*. B. sinica cell cluster borders are outlined by *Chloroflexi* bacteria, with (**B**) *Ca*. B. sinica S-layer enveloping the anammox bacterial cells and (**C**) presenting at the edge of the biofilm. **D** Normalised fluorescence intensity line profile along the dashed line in A (line width = 18) showing the change in interaction between S-layer protein (green), *Chloroflexi* (blue) and anammox bacteria (red) from the edge (location 1) to the interior (location 6) of the biofilm. **E** 3-D reconstructed image of **A**. **F** 3-D reconstructed anammox biofilm topology in which S-layer protein binds only to the border of the anammox cell clusters as indicated by white arrows. Scale bars indicate 10 µm.

The spatial distribution of the dominant extracellular S-layer protein, BROSI_A1236 in the biofilm, relative to *Chloroflexi* and *Ca*. B. sinica is illustrated by the thin sliced anammox biofilm sectioned following FISH and immunostaining (Fig. 4). The specificity of the BROSI_A1236 antibody is indicated from western blot analysis of the crude EPS extract (SI Fig. 1) by an intense interaction between the antibody and the 170/200 kDa protein doublet that was previously attributed to BROSI_A1236 of *Ca*. B. sinica [35].

Three distinct S-layer protein binding regions (green) relative to *Ca*. B. sinica cells (red) were visible across the anammox biofilm section (Fig. 4A–E, SI Figs. 3,  4). The antibody was bound to the envelope of *Ca*. B. sinica cells within the biofilm (dashed box B, Fig. 4A, B). This is further demonstrated by overlapping fluorescent signals for the S-layer antibody and *Ca*. B. sinica FISH probes in the fluorescence intensity line profile in this region (Fig. 4D, region 3, red and green lines respectively). This observation provides further evidence that the anammox extracellular protein is an S-layer protein [35].

The S-layer protein also appeared at the edge of the biofilm, along with *Chloroflexi* and where *Ca*. B. sinica is largely absent (Fig. 4A, dashed box C and Fig. 4C). In this region, the S-layer protein occupied the space around the *Chloroflexi* (Fig. 4A, regions 1 and 4 of line 4D; SI Fig. 5), which is in contrast to the polysaccharides that only coat the surface of the *Chloroflexi* (Fig. 3H, I). The Diamond sequence aligner run against all *Chloroflexi* returned no match for BROSI_A1236 to any *Chloroflexi* MAGs. Furthermore, no other major microbial population was observed at the edge of the biofilm (SI Fig. 6).

Additionally, in another region close to the surface of the biofilm (dashed box F of Fig. 4E), some *Ca*. B. sinica cells were not labelled by the S-layer protein antibody. There is, however, an intense S-layer protein antibody signal at the border of this cluster (indicated by white arrows in Fig. 4F). This demonstrates that the antibody is selective and that the S-layer proteins may not necessarily be present on the surface of all anammox cells in the community.

### Anammox S-layer protein coincides with *Chloroflexi* at the junctions of *Chloroflexi* structural networks

**Fig. 5:**
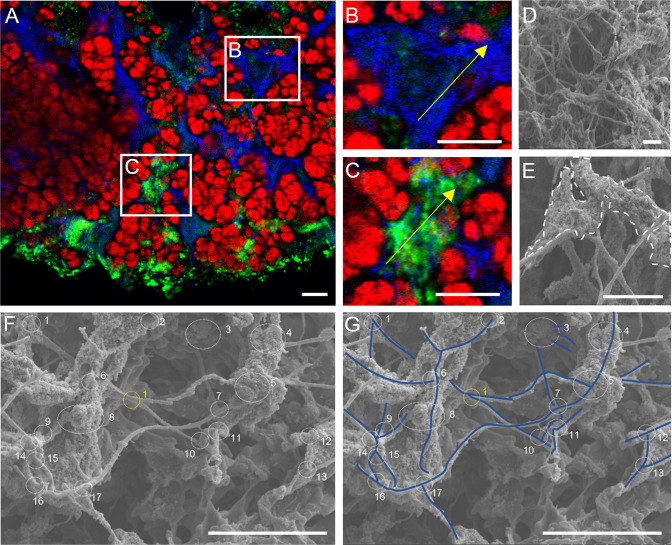
Confocal laser scanning micrographs (**A**, **B**, **C**) and scanning electron micrographs (**D**, **E**, **F**, **G**) showing (**A**) filamentous *Chloroflexi* cell (labelled by *Chloroflexi*-phylum probes CFX1223 and GNSB941, blue) demarcating anammox bacterial cell clusters (labelled by *Ca*. B. sinica FISH probe Bsi630, red) in cross-linked network with (**B**, **D**) weak and (**C**, **E**) strong S-layer protein (labelled by S-layer protein antibody and visualised with Alexa Fluor 488-labelled goat anti-rabbit IgG, green) associations. Regions marked by white dotted lines in **E** are likely S-layer proteins that assemble *Chloroflexi* cells in the structural lattice. **F**, **G** show filamentous *Chloroflexi* cells forming a cross-linked framework with junctions (i.e., intersections formed by two or more filamentous *Chloroflexi* cells) that are coated and non-coated with extracellular matrix biopolymer. Blue solid lines in G trace *Chloroflexi* filaments in the biofilm. Scale bars indicate 10 μm in **A**, **B**, **C** and 5 μm in **D**, **E**, **F**, **G**. Yellow lines in images **B**, **C** indicate regions where fluorescence intensity line profiles of interactions between S-layer protein and *Chloroflexi* cells were plotted in SI Fig. 7 Figure 5A was imaged at the layer distal from the surface of the anammox biofilm cross-section. Therefore, most of the antibody staining was observed at the edge of the biofilm.

The association between *Chloroflexi* and the S-layer protein also depends on the proximity to the biofilm surface. *Chloroflexi* and S-layer proteins coexist towards the outer edge of the biofilm (Fig. 4C), as indicated by the elevated fluorescent signals at the biofilm surface (Fig. 4D, region 1, green and blue lines). The S-layer protein antibody signal also coincides with the *Chloroflexi* at junctions in the *Chloroflexi* network (Fig. 5A–C), particularly towards the outer edge (Fig. 5C) rather than the interior of the biofilm (Fig. 5B and SI Fig. 7).

The EPS that overlapped with *Chloroflexi* cells, as shown in SEM images (Fig. 3J, K), is proposed to be S-layer protein, as demonstrated by immunofluorescence staining (Fig. 5B–E). The SEM images showed filamentous bacteria embedded by an extracellular matrix and forming a 3-D scaffold (Figs. 3K, 5). The extracellular matrix coating the *Chloroflexi* at the nodes of the filamentous framework is denoted by white dotted lines in Fig. 5E. This was observed across multiple regions in SEM images (SI Fig. 8). More than 80% of the *Chloroflexi* junctions were coated by the extracellular matrix biopolymer, as determined by tracing the filamentous network and identifying junctions as either matrix-associated or non-matrix-associated (see Fig. 5F, G for the representative images; i.e., 105 out of 119 junctions were matrix-associated, as determined by several images. Refer to SI Fig. 8 for remaining images).

### Potential pathways for succession of BROSI_A1236 from surface protein to EPS

**Fig. 6:**
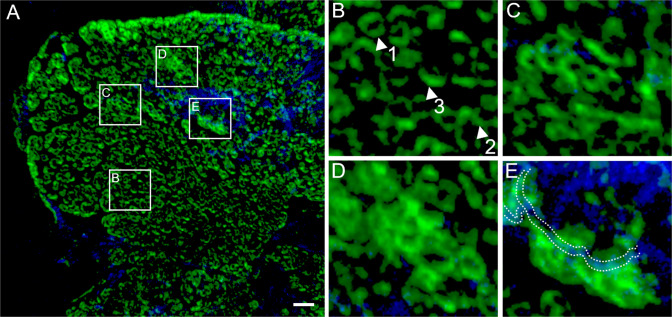
Different morphologies of S-layer protein (250x dilution, identified using S-layer protein antibody and visualised with Alexa Fluor 488-labelled goat anti-rabbit IgG, green) relative to *Chloroflexi* (identified using *Chloroflexi*-phylum probes CFX1223 and GNSB941), suggesting (**A**) S-layer protein transitions from enveloping cell surface to an EPS and matrix stabilising agent. **B** S-layer protein expressed as surface layer protein by anammox bacteria, covering the cells as a complete (white arrowhead 1), partially (white arrowhead 2) or fully opened (white arrowhead 3) ring structure. **C** These closed and partially closed rings then start to coalesce, **D** Consolidate further into an aggregate of S-layer proteins, and (**E**) finally start aggregating around *Chloroflexi* cells (the *Chloroflexi* FISH probes labelled cells are highlighted with white dotted lines). Scale bar indicates 10 μm.

The anammox biofilm cross-section in Fig. 6A highlights the various forms in which BROSI_A1236 exists in the biofilm, relative to the *Chloroflexi*. The S-layer protein initially forms well-defined closed or partially closed surface protein rings (Fig. 6B) that coalesce to form extended chains (Fig. 6C). These subsequently develop into aggregates of larger assemblages within anammox clusters (Fig. 6D) and finally form assemblages that coat the *Chloroflexi* cells (Fig. 6E). In the larger BROSI_A1236 assemblages surrounding *Chloroflexi* cells, traces of the original BROSI_A1236 ring structure can still be discerned (Fig. 6D, E). The same image including a *Ca*. B. sinica signal indicates that *Ca*. B. sinica co-occurs with BROSI_A1236 aggregates in the internal regions of the biofilm as the latter begins to coat the *Chloroflexi* cells (SI Fig. 9). Moreover, the same traces of the surface protein rings coating the *Chloroflexi* in the absence of *Ca*. B. sinica, are also visible in the border regions of the biofilm (Fig. 4C).

## Discussion

This study reveals a strong association of the S-layer protein BROSI_A1236 with the envelope of *Ca*. B. sinica cells. The S-layer protein also contributes to a thick extracellular matrix layer at the edge of the biofilm surface and in close proximity to *Chloroflexi*. The filamentous *Chloroflexi* assemble into a cross-linked network throughout and at the surface of the anammox biofilm, and the *Ca*. B. sinica S-layer protein is present at network junctions. The intensity of the western blot signal for antibody binding to the high molecular weight protein doublet in the crude EPS that was previously identified as the surface protein, BROSI_A1236 of *Ca*. B. sinica, was high relative to other proteins [35]. Low level binding of the antibody to lower molecular weight proteins is visible in the western blot, and it is possible that some of the signals in the immunofluorescence microscopy images result from non-specific binding (SI Fig. 1). Nonetheless, overlaying the general protein stain (i.e., SYPRO Ruby) with the BROSI_A1236 immunofluorescence (SI Fig. 10) shows that, while BROSI_A1236 is abundant, it represents only a small fraction of the protein signal on the biofilm cross-section, further indicating antibody specificity (approximately 20% biovolume). This, along with the inability of any *Chloroflexi* to express BROSI_A1236 and the absence of any other major microbial populations within the regions where *Chloroflexi* and the surface protein co-localise at the biofilm borders (SI Fig. 6), suggest that *Chloroflexi* has acquired the S-layer protein that is secreted by *Ca*. B. sinica. Hence, the surface layer protein likely facilitates another species to form a structural scaffold and act as an adhesive as well as EPS. The *Chloroflexi* network in turn accommodates clusters of anammox cells. Thus, *Ca*. B. sinica secretion of the S-layer protein enables the organisation of a cooperating community into a biofilm structural matrix for the benefit of the whole community.

We have demonstrated that the S-layer protein is secreted by the *Ca*. B. sinica and transported to the biofilm edge; however, the means by which this is achieved is not clear. No paracrystalline structures were observed in the matrix at the edge of biofilm, although this could possibly be due to insufficient resolution of the SEM used here (Fig. 5E–G). Nonetheless, the transport of the S-layer protein from the cell surface to the biofilm surface likely involves the S-layer protein transitioning between different phases or states, as illustrated in Fig. 6. Several mechanisms exist by which the S-layer protein could achieve this. The anammox biofilm S-layer protein possesses intrinsically disordered repeat domains (IDRs) at the C-terminus, which could facilitate phase transitions and the passaging of the S-layer protein through the matrix [45, 46]. Alternatively, the glycosylated S-layer protein could adopt different proteoforms throughout the biofilm (e.g., glycosylated or unglycosylated forms) to facilitate the transition of S-layer protein to cells in the biofilm and its transformation into EPS [47–49].

**Fig. 7:**
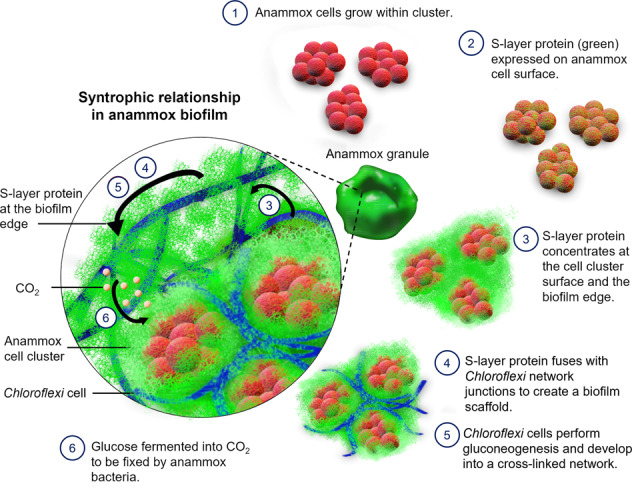
A proposed model of *Ca*. B. sinica coordinated assembly of filamentous *Chloroflexi* cells into anammox biofilm structural scaffold through excretion of S-layer protein, BROSI_A1236 as an EPS.

The heterotrophic *Chloroflexi* metabolise anammox EPS [21]. The close association between *Chloroflexi* cells and the S-layer protein described in this study suggests that the EPS metabolised by *Chloroflexi* could be the anammox S-layer protein (Fig. 5). Con A staining showed a strong overlap of polysaccharides with *Chloroflexi* cells within internal anammox cell cluster channels, and at the biofilm surface (Fig. 3). This indicates that the polysaccharide is located on the cellular surface of the *Chloroflexi* (i.e., as capsular), as opposed to the extracellular biopolymer, which encompasses the *Chloroflexi* cells, as illustrated in the SEM images (Fig. 5E–G). Based on the experimental spatial localisation of the S-layer protein, the anammox exoprotein is proposed to enable anaerobic growth, gluconeogenesis and subsequent expression of the polysaccharide capsule of *Chloroflexi* [50, 51]. In addition to benefitting *Ca*. B. sinica by establishing a biofilm scaffold that accommodates necessary synergistic relationships, the carbon dioxide produced from fermentation of the S-layer protein by *Chloroflexi* would also be available to the anammox bacteria for carbon fixation via the Wood-Ljungdahl pathway for subsequent biomass precursor production [52]. The S-layer protein could therefore function as a “public-good” exopolymer and coordinate the organisation of key populations in a polymicrobial biofilm (Fig. 7) [53, 54].

Mixed species biofilms are the dominant microbial life form in environmental and industrial settings [14, 55]. However, there are few examples where the distribution of specific EPS has been described relative to key microbial populations. To the authors’ best knowledge, the anammox biofilm results provided here constitute the first report of such a structure-function relationship for industrial or environmental biofilms. The S-layer protein was, however, previously observed on anammox bacterial cell surfaces, and it has been detected in the extracellular extracts from anammox biofilms enriched in different anammox bacterial species [56]. The inability to confirm its function as an EPS arose from a lack of means to visualise the protein (Figs. 1B, 3E, and SI Fig. 10).

This study describes how EPS may mediate structure, composition and function in a model mixed-species biofilm [57]. We propose that anammox cells use the S-layer protein to promote cellular adhesion and microcolony formation within a structural scaffold established by the second most abundant population (i.e., *Chloroflexi*) in the biofilm community. The spatial location and succession of the S-layer suggests it has a moonlighting role as a *Chloroflexi* cross-linking network and stabilising material as well as a driver for biofilm community assembly (Fig. 7). High co-enrichments of *Chloroflexi* and *Ca*. Brocadia in anammox bioprocesses, and a scaffold-building function for *Chloroflexi* in providing a network for cells and microcolonies to adhere and aggregate have been described in activated sludge and anammox biofilms [23, 58–62]. These findings may have direct implications in activated sludge microbiology [22]. Following the first description of an anammox S-layer glycoprotein [56], BROSI_A1236 and its homologues have been identified as a common feature of anammox biofilms and, more recently, as an EPS [35, 45, 63]. There is increasing interest in all aspects of this glycoprotein, including understanding how it is glycosylated [64], and its function as an EPS. While our work provides additional insight into its function in enabling community assembly, it also suggests a key role for S-layer proteins in the formation of biofilms. Overall, this work highlights the importance of the spatial organisation for syntrophic relationships within mixed species microbial biofilms [55, 65] and may underpin strategies for optimising anammox bioprocesses, such as increasing biofilm stability or reducing bioprocess start-up times [45, 66].

## Supplementary information

Supplementary material

## Data Availability

The data supports the findings in this study are available within the article as well as the Supplementary material. Raw data is available upon request.

## References

[1] Jayathilake PG, Jana S, Rushton S, Swailes D, Bridgens B, Curtis T (2017). Extracellular polymeric substance production and aggregated bacteria colonization influence the competition of microbes in biofilms. Front Microbiol.

[2] Flemming H-C, Neu TR, Wingender J (eds). The Perfect Slime: Microbial Extracellular Polymeric Substances (EPS). IWA Publishing, London, 2016.

[3] Morales-García AL, Bailey RG, Jana S, Burgess JG (2019). The role of polymers in cross-kingdom bioadhesion. Philos Trans R Soc Lond B Biol Sci.

[4] Davey ME, O’Toole GA (2000). Microbial biofilms: From ecology to molecular genetics. Microbiol Mol.

[5] Peters BM, Jabra-Rizk MA, O’May GA, Costerton JW, Shirtliff ME (2012). Polymicrobial interactions: Impact on pathogenesis and human disease. Clin Microbiol Rev.

[6] Karygianni L, Ren Z, Koo H, Thurnheer T (2020). Biofilm matrixome: Extracellular components in structured microbial communities. Trends Microbiol.

[7] Morris BEL, Henneberger R, Huber H, Moissl-Eichinger C (2013). Microbial syntrophy: Interaction for the common good. FEMS Microbiol Rev.

[8] Decho AW, Gutierrez T (2017). Microbial extracellular polymeric substances (EPSs) in ocean systems. Front Microbiol.

[9] Boleij M, Seviour T, Wong LL, van Loosdrecht MCM, Lin Y (2019). Solubilization and characterization of extracellular proteins from anammox granular sludge. Water Res.

[10] Kim D, Barraza JP, Arthur RA, Hara A, Lewis K, Liu Y (2020). Spatial mapping of polymicrobial communities reveals a precise biogeography associated with human dental caries. Proc Natl Acad Sci USA.

[11] Sadiq FA, Burmølle M, Heyndrickx M, Flint S, Lu W, Chen W (2021). Community-wide changes reflecting bacterial interspecific interactions in multispecies biofilms. Crit Rev Microbiol.

[12] Liu W, Jacquiod S, Brejnrod A, Russel J, Burmølle M, Sørensen SJ (2019). Deciphering links between bacterial interactions and spatial organization in multispecies biofilms. ISME J.

[13] Bridier A, Le Coq D, Dubois-Brissonnet F, Thomas V, Aymerich S, Briandet R (2011). The spatial architecture of *Bacillus subtilis* biofilms deciphered using a surface-associated model and in situ imaging. PLOS One.

[14] Lee KWK, Periasamy S, Mukherjee M, Xie C, Kjelleberg S, Rice SA (2014). Biofilm development and enhanced stress resistance of a model, mixed-species community biofilm. ISME J.

[15] Myszka K, Czaczyk K (2009). Characterization of adhesive exopolysaccharide (EPS) produced by *Pseudomonas aeruginosa* under starvation conditions. Curr Microbiol.

[16] Harimawan A, Ting YP (2016). Investigation of extracellular polymeric substances (EPS) properties of *P. aeruginosa* and *B. subtilis* and their role in bacterial adhesion. Colloids Surf B.

[17] Yang X-R, Li H, Nie S-A, Su J-Q, Weng B-S, Zhu G-B (2015). Potential contribution of anammox to nitrogen loss from paddy soils in Southern China. Appl Environ Microbiol.

[18] Kartal B, van Niftrik L, Keltjens JT, Op den Camp HJM, Jetten MSM Chapter 3 - Anammox—Growth physiology, cell biology, and metabolism. In: Poole RK, editor. Adv Microb Physiol. 60: Academic Press; 2012. p. 211–62. 10.1016/B978-0-12-398264-3.00003-6 22633060

[19] Lu Y, Natarajan G, Nguyen TQN, Thi SS, Arumugam K, Seviour TW, et al. Species level enrichment of AnAOB and associated growth morphology under the effect of key metabolites. bioRxiv. 2020. 2020.02.04.934877

[20] Gonzalez-Gil G, Sougrat R, Behzad AR, Lens PN, Saikaly PE (2015). Microbial community composition and ultrastructure of granules from a full-scale anammox reactor. Micro Ecol.

[21] Kindaichi T, Yuri S, Ozaki N, Ohashi A (2012). Ecophysiological role and function of uncultured *Chloroflexi* in an anammox reactor. Water Sci Technol.

[22] Qin Y, Han B, Cao Y, Wang T (2017). Impact of substrate concentration on anammox-UBF reactors start-up. Bioresour Technol.

[23] Chen Z, Meng Y, Sheng B, Zhou Z, Jin C, Meng F (2019). Linking exoproteome function and structure to anammox biofilm development. Environ Sci Technol.

[24] Ali M, Shaw DR, Albertsen M, Saikaly PE (2020). Comparative genome-centric analysis of freshwater and marine ANAMMOX cultures suggests functional redundancy in nitrogen removal processes. Front Microbiol.

[25] Jia F, Yang Q, Liu X, Li X, Li B, Zhang L (2017). Stratification of extracellular polymeric substances (EPS) for aggregated anammox microorganisms. Environ Sci Technol.

[26] Hou X, Liu S, Zhang Z (2015). Role of extracellular polymeric substance in determining the high aggregation ability of anammox sludge. Water Res.

[27] Feng C, Lotti T, Lin Y, Malpei F (2019). Extracellular polymeric substances extraction and recovery from anammox granules: Evaluation of methods and protocol development. Chem Eng J.

[28] Lotti T, Carretti E, Berti D, Montis C, Del Buffa S, Lubello C (2019). Hydrogels formed by anammox extracellular polymeric substances: Structural and mechanical insights. Sci Rep..

[29] Jakubovics NS, Goodman SD, Mashburn-Warren L, Stafford GP, Cieplik F (2021). The dental plaque biofilm matrix. Periodontology 2000.

[30] Ðapa T, Leuzzi R, Ng YK, Baban ST, Adamo R, Kuehne SA (2013). Multiple factors modulate biofilm formation by the anaerobic pathogen *Clostridium difficile*. J Bacteriol.

[31] Honma K, Inagaki S, Okuda K, Kuramitsu HK, Sharma A (2007). Role of a *Tannerella forsythia* exopolysaccharide synthesis operon in biofilm development. Micro Pathog.

[32] Li X-R, Du B, Fu H-X, Wang R-F, Shi J-H, Wang Y (2009). The bacterial diversity in an anaerobic ammonium-oxidizing (anammox) reactor community. Syst Appl Microbiol.

[33] Cho S, Takahashi Y, Fujii N, Yamada Y, Satoh H, Okabe S (2010). Nitrogen removal performance and microbial community analysis of an anaerobic up-flow granular bed anammox reactor. Chemosphere.

[34] Morgenroth E, Sherden T, Van Loosdrecht MCM, Heijnen JJ, Wilderer PA (1997). Aerobic granular sludge in a sequencing batch reactor. Water Res.

[35] Wong LL, Natarajan G, Boleij M, Thi SS, Winnerdy FR, Mugunthan S (2020). Extracellular protein isolation from the matrix of anammox biofilm using ionic liquid extraction. Appl Microbiol Biotechnol.

[36] Law Y, Kirkegaard RH, Cokro AA, Liu X, Arumugam K, Xie C (2016). Integrative microbial community analysis reveals full-scale enhanced biological phosphorus removal under tropical conditions. Sci Rep.

[37] Ondov BD, Bergman NH, Phillippy AM (2011). Interactive metagenomic visualization in a Web browser. BMC Bioinform.

[38] Liu X, Arumugam K, Natarajan G, Seviour TW, Drautz-Moses DI, Wuertz S (2018). Draft genome sequence of a *Candidatus* brocadia bacterium enriched from activated sludge collected in a tropical climate. Genome Announc.

[39] Price MN, Dehal PS, Arkin AP (2010). FastTree 2-approximately maximum-likelihood trees for large alignments. PloS One.

[40] Arkin AP, Cottingham RW, Henry CS, Harris NL, Stevens RL, Maslov S (2018). KBase: The United States Department of Energy Systems Biology Knowledgebase. Nat Biotechnol.

[41] Parks DH, Imelfort M, Skennerton CT, Hugenholtz P, Tyson GW (2015). CheckM: Assessing the quality of microbial genomes recovered from isolates, single cells, and metagenomes. Genome Res.

[42] Buchfink B, Reuter K, Drost H-G (2021). Sensitive protein alignments at tree-of-life scale using DIAMOND. Nat Methods.

[43] Buchfink B, Xie C, Huson DH (2015). Fast and sensitive protein alignment using DIAMOND. Nat Methods.

[44] Daims H, Nielsen JL, Nielsen PH, Schleifer K-H, Wagner M (2001). In situ characterization of Nitrospira-like nitrite-oxidizing bacteria active in wastewater treatment plants. Appl Environ Microbiol.

[45] Seviour T, Wong LL, Lu Y, Mugunthan S, Yang Q, Shankari UDOCS (2020). Phase transitions by an abundant protein in the anammox extracellular matrix mediate cell-to-cell aggregation and biofilm formation. mBio.

[46] Protter DSW, Rao BS, Van Treeck B, Lin Y, Mizoue L, Rosen MK (2018). Intrinsically disordered regions can contribute promiscuous interactions to RNP granule assembly. Cell Rep.

[47] Fulton KM, Smith JC, Twine SM (2016). Clinical applications of bacterial glycoproteins. Expert Rev Proteom.

[48] Upreti RK, Kumar M, Shankar V (2003). Bacterial glycoproteins: Functions, biosynthesis and applications. Proteomics.

[49] van Teeseling MCF, Maresch D, Rath CB, Figl R, Altmann F, Jetten MSM (2016). The S-layer protein of the anammox bacterium *Kuenenia stuttgartiensis*s is heavily O-glycosylated. Front Microbiol.

[50] McGonigle JM, Lang SQ, Brazelton WJ, Parales RE (2020). Genomic evidence for formate metabolism by *Chloroflexi* as the key to unlocking deep carbon in lost city microbial ecosystems. Appl Environ Microbiol.

[51] Vuillemin A, Kerrigan Z, D’Hondt S, Orsi WD (2020). Exploring the abundance, metabolic potential and gene expression of subseafloor *Chloroflexi* in million-year-old oxic and anoxic abyssal clay. FEMS Microbiol Ecol.

[52] Kartal B, de Almeida NM, Maalcke WJ, Op den Camp HJ, Jetten MS, Keltjens JT (2013). How to make a living from anaerobic ammonium oxidation. FEMS Microbiol Rev.

[53] Loera-Muro A, Guerrero-Barrera A, Tremblay DNY, Hathroubi S, Angulo C (2021). Bacterial biofilm-derived antigens: A new strategy for vaccine development against infectious diseases. Expert Rev Vaccines.

[54] Hobley L, Harkins C, MacPhee CE, Stanley-Wall NR (2015). Giving structure to the biofilm matrix: An overview of individual strategies and emerging common themes. FEMS Microbiol Rev.

[55] Elias S, Banin E (2012). Multi-species biofilms: Living with friendly neighbors. FEMS Microbiol Rev.

[56] Teeseling MCFV, Almeida NMD, Klingl A, Speth DR, Camp HJMOD, Rachel R (2014). A new addition to the cell plan of anammox bacteria: *Candidatus* Kuenenia stuttgartiensis has a protein surface layer as the outermost layer of the cell. J Bacteriol.

[57] Paula AJ, Hwang G, Koo H (2020). Dynamics of bacterial population growth in biofilms resemble spatial and structural aspects of urbanization. Nat Commun.

[58] Kragelund C, Caterina L, Borger A, Thelen K, Eikelboom D, Tandoi V (2007). Identity, abundance and ecophysiology of filamentous *Chloroflexi* species present in activated sludge treatment plants. FEMS Microbiol Ecol.

[59] Nierychlo M, Miłobędzka A, Petriglieri F, McIlroy B, Nielsen PH, McIlroy SJ. The morphology and metabolic potential of the *Chloroflexi* in full-scale activated sludge wastewater treatment plants. FEMS Microbiol Ecol. 2018;95. 10.1093/femsec/fiy228 30476032

[60] Kragelund C, Thomsen TR, Mielczarek AT, Nielsen PH (2011). Eikelboom’s morphotype 0803 in activated sludge belongs to the genus *Caldilinea* in the phylum *Chloroflexi*. FEMS Microbiol Ecol.

[61] Zhang J, Miao Y, Zhang Q, Sun Y, Wu L, Peng Y (2020). Mechanism of stable sewage nitrogen removal in a partial nitrification-anammox biofilm system at low temperatures: Microbial community and EPS analysis. Bioresour Technol.

[62] Björnsson L, Hugenholtz P, Tyson GW, Blackall LL (2002). Filamentous *Chloroflexi* (green non-sulfur bacteria) are abundant in wastewater treatment processes with biological nutrient removal. Microbiology.

[63] Boleij M, Pabst M, Neu TR, van Loosdrecht MCM, Lin Y (2018). Identification of glycoproteins isolated from extracellular polymeric substances of full-scale anammox granular sludge. Environ Sci Technol.

[64] Pabst M, Grouzdev DS, Lawson CE, Kleikamp HBC, de Ram C, Louwen R (2022). A general approach to explore prokaryotic protein glycosylation reveals the unique surface layer modulation of an anammox bacterium. ISME J.

[65] Berlanga M, Guerrero R (2016). Living together in biofilms: The microbial cell factory and its biotechnological implications. Micro Cell Fact.

[66] Liu T, Tian R, Li Q, Wu N, Quan X (2021). Strengthened attachment of anammox bacteria on iron-based modified carrier and its effects on anammox performance in integrated floating-film activated sludge (IFFAS) process. Sci Total Environ.

