# Supplementary material for: Surface-layer protein is a public-good matrix exopolymer for microbial community organisation in environmental anammox biofilms
Source: ISME J. 2023 Mar 4;17(6):803–12. doi: 10.1038/s41396-023-01388-y (PMC10202920; doi:10.1038/s41396-023-01388-y)
Supplement: Supplementary file 1 — Supplementary material [file 41396_2023_1388_MOESM1_ESM.pdf]

Supplementary information

Surface-layer protein is a public-good matrix exopolymer for microbial community organisation in environmental anammox biofilms

Lan Li Wong[1,2], Yang Lu[3], James Chin Shing Ho[1], Sudarsan Mugunthan[1], Yingyu Law[1], Patricia Conway[1,4] , Staffan Kjelleberg[1,2,4], Thomas Seviour[1,5]

[1] Singapore Centre for Environmental Life Sciences Engineering, Nanyang Technological University, Singapore, 637551, Singapore

[2] School of Biological Sciences, Nanyang Technological University, Singapore, 637551, Singapore

[3] The Australian Centre for Ecogenomics, School of Chemistry and Molecular Biosciences, The University of Queensland, St Lucia, Queensland, Australia 4072

[4] School of Biological, Earth and Environmental Sciences, University of New South Wales Sydney, 2052, Australia

[5] WATEC Aarhus University Centre for Water Technology, Universitetsbyen 36, Bldg 1783, 8000 Aarhus, Denmark

*Correspondence to: twseviour@bce.au.dk; laskjelleberg@ntu.edu.sg

This PDF file includes:

Supplementary figures 1 to 8

Supplementary Tables 1 and 2

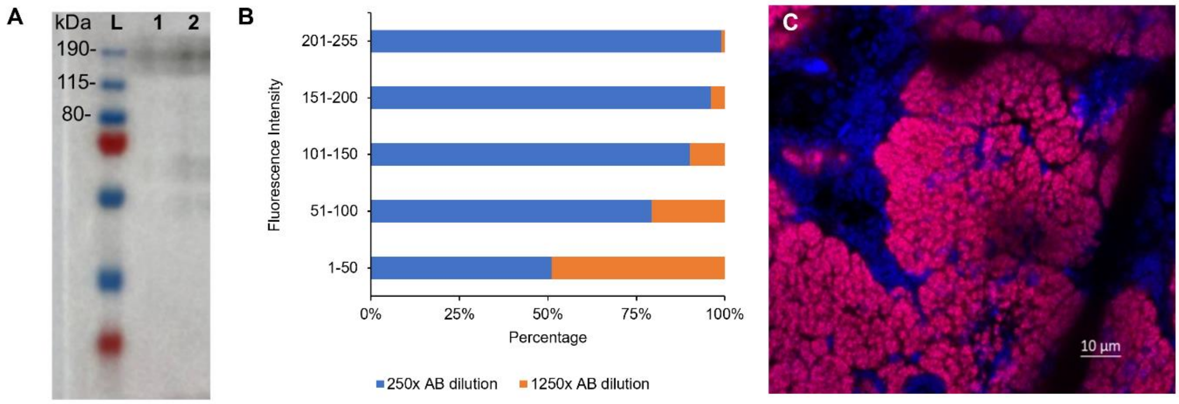

Supplementary figure 1: **S-layer protein primary antibody validation.** (A) Immunoblotting validation by western blot showing positive blot of high molecular weight S-layer protein, BROSI_A1236 doublet (170/200kDa bands) against S-layer protein antibody (AB). (Lane L: protein ladder, lanes 1 and 2 were loaded with 0.2 µg and 0.4 µg of crude anammox EPS extract respectively) (B) Bar chart showing concentration-dependent staining using S-layer protein antibody (visualised using Alexa Fluor 488-labelled goat anti-rabbit IgG secondary antibody) on an anammox biofilm thin section. (C) Confocal laser scanning micrograph showing no visible staining with Alexa Fluor 488-labelled goat anti-rabbit IgG, secondary antibody (green) in the absence of an S-layer protein, BROSI_A1236 primary antibody on 10 µm anammox granular sludge thin section counterstained with *Ca.* B. sinica-specific (red) and general bacterial (EUB-338 mix) (blue) FISH probes. Scale bar indicates 10 µm.

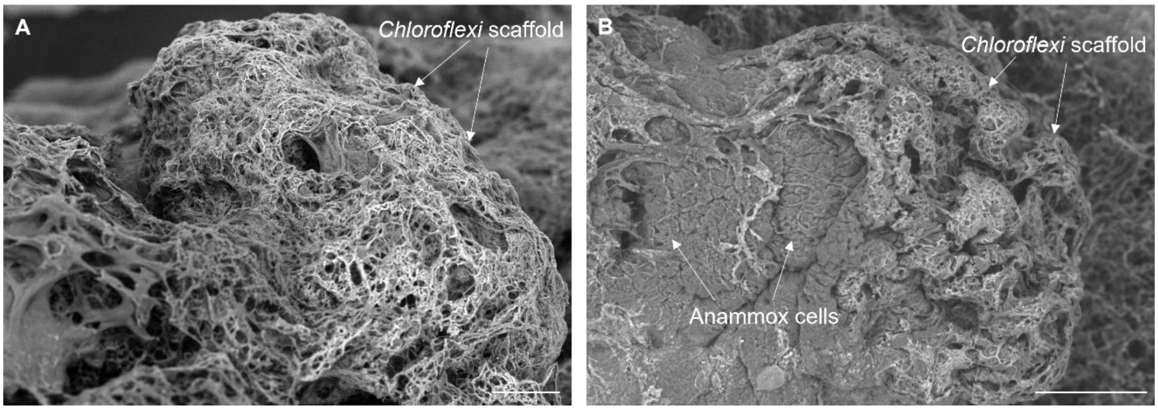

Supplementary figure 2: Filamentous *Chloroflexi* cells form scaffold in anammox biofilm as a cross-linked network. Variable pressure scanning electron micrographs (VP-SEM) showing (A) filamentous *Chloroflexi* cross-linked network and (B) the cross-linked network surrounding anammox cells of anammox granular sludge. The images were taken under low pressure conditions. Scale bars indicate 50 μm.

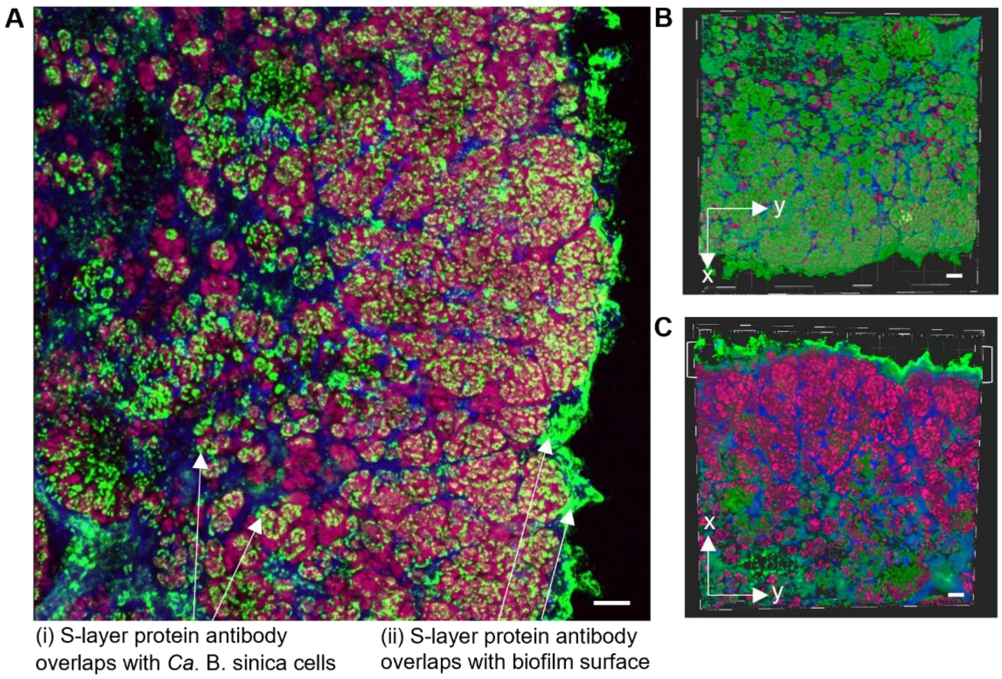

Supplementary figure 3: **Anammox surface layer protein, BROSI_A1236 forms envelope around anammox cell and concentrates at the edges of anammox cells clusters.** (A) z-stack confocal laser scanning microscopy (CLSM) images (10 µm, consisting of 13 layers) of anammox biofilm stained with S-layer protein antibody (200x dilution) and visualised with Alexa Fluor 488-labelled goat anti-rabbit IgG (green), showing the S-layer protein coats (i) the surface of the anammox bacterial cells as well as (ii) the biofilm edge. EUB-338 I, II and III mix FISH probes (blue) as general bacterial marker and *Ca.* B. sinica-specific FISH probe (red) were used. (B-C) 3-D reconstructed image of Figure SI 3A shows the accumulation of the S-layer protein at the biofilm edge. The top (B) and the bottom (C) views of the biofilm are depicted. Scale bars indicate 10 µm.

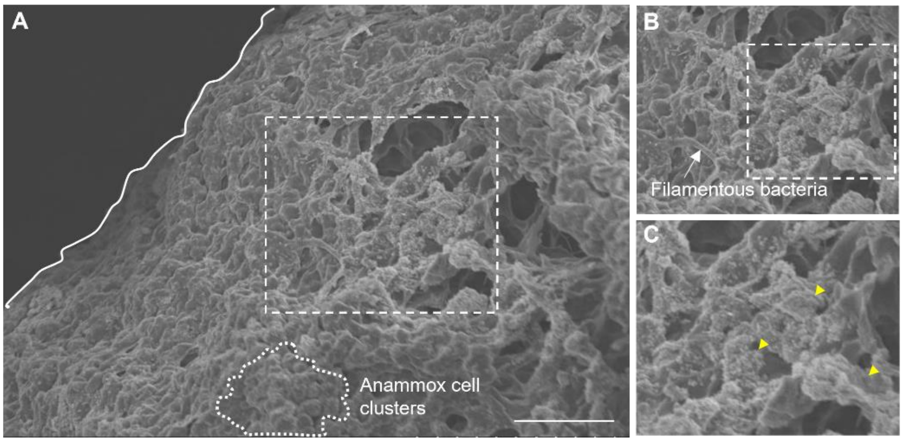

Supplementary figure 4: **Distribution of *Ca.* B. sinica, S-layer protein, BROSI_A1236 and general bacteria on anammox granules.** Scanning electron micrograph of (A) freeze dried anammox granule showing uneven biofilm surface delineated by solid white line at the edge of biofilm. Scale bar represents 20 μm. (B) The enlarged view of the white dashed box in (A) shows filamentous bacteria interspersed with anammox cells. (C) The enlarged view of the white dashed box in (B) shows off-white coloured coatings (yellow arrowheads) on anammox bacteria cells that are likely made up of S-layer protein.

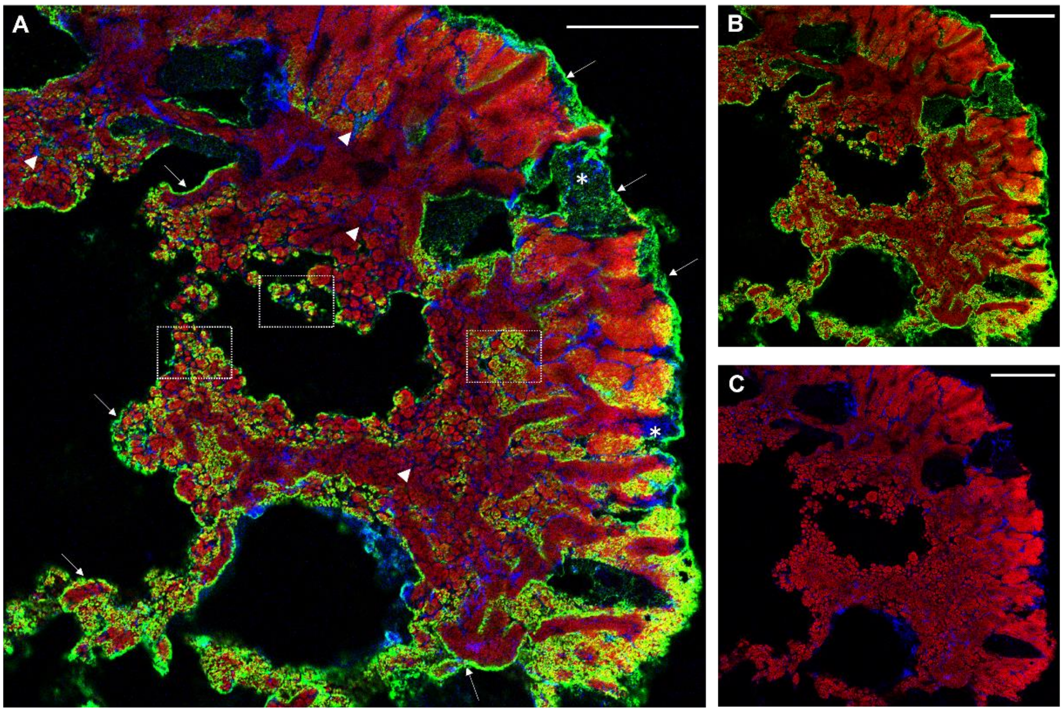

Supplementary figure 5: **S-layer protein, BROSI_A1236 occupies space around the *Chloroflexi* in anammox biofilm.** (A) Confocal laser scanning micrograph shows an anammox biofilm thin section with *Chloroflexi* (labelled by general *Chloroflexi* phylum CFX1223 and GNSB941 FISH probes, blue) demarcating anammox cell clusters (positively stained by *Ca.* B. sinica specific Bsi630 FISH probe (red)) at the internal channels between *Ca.* B. sinica cells (arrowheads) and at the periphery of the biofilm (asterisk). The distribution of S-layer protein (immunostaining at 250x dilution, visualised with Alexa Fluor 488-labelled goat anti-rabbit IgG, green) on anammox cell cluster surfaces (white dotted box) and at the biofilm edges (white arrows) is overlaid. Overlay images of *Ca.* B. sinica cells and (B) S-layer protein or (C) *Chloroflexi*. Scale bars indicate 20 µm.

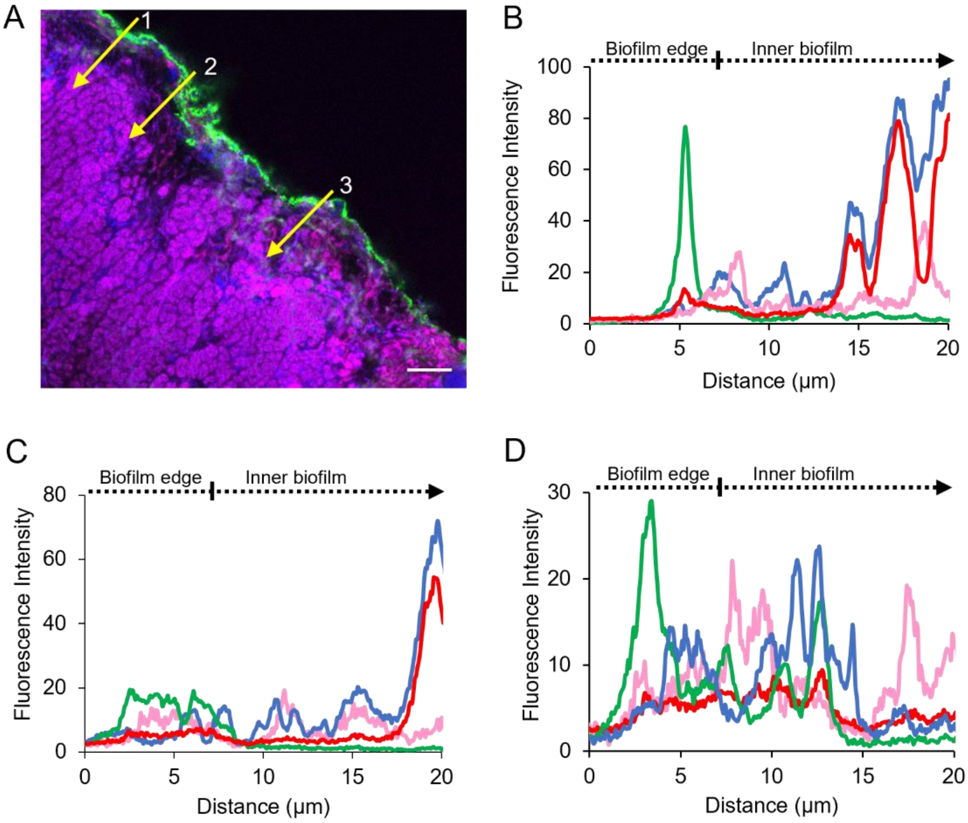

Supplementary figure 6: **Low fluorescence signal of non-anammox bacterial cells along the biofilm edge suggests that *Chloroflexi* acquires network stabilising material (i.e., likely S-layer protein) that is secreted by anammox cells.** (A) Confocal laser scanning micrograph shows an anammox biofilm thin section labelled with S-layer protein antibody (250x dilution) and visualised with Alexa Fluor 405-labelled goat anti-rabbit IgG (green), general bacterial marker, EUB-338 I, II and III mix (blue), *Chloroflexi* phylum CFX1223 and GNSB941 FISH probes (magenta) and *Ca.* B. sinica FISH probe (red). (B-D) The fluorescence intensity line profile along the arrows (1-3) indicated in the confocal image (line width = 18, arrows pointing towards biofilm interior) shows fluorescence signals of S-layer protein (green), anammox bacteria (red), *Chloroflexi* cells (magenta) and all bacteria (blue). Figures B-D show high S-layer protein fluorescence signal (green) at the edge of the biofilm as opposed to cells that are non-anammox and *Chloroflexi*. The relative general bacterial fluorescence intensity (blue) across 5-17 µm is less than 20 after subtraction of anammox (red) and *Chloroflexi* (magenta) cells.

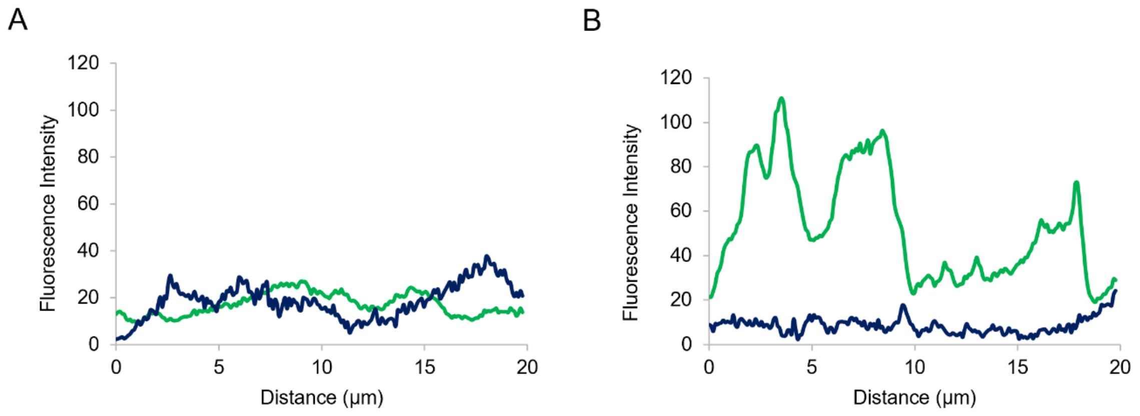

Supplementary figure 7: **S-layer protein displays depth-dependent association with *Chloroflexi* cells.** Fluorescence intensity line profile (line width = 18) of the lines indicated in Figure 5B and C demonstrating distinct interactions between S-layer protein (green) and *Chloroflexi* (blue) at the (A) interior and at the (B) surface of the biofilm, respectively.

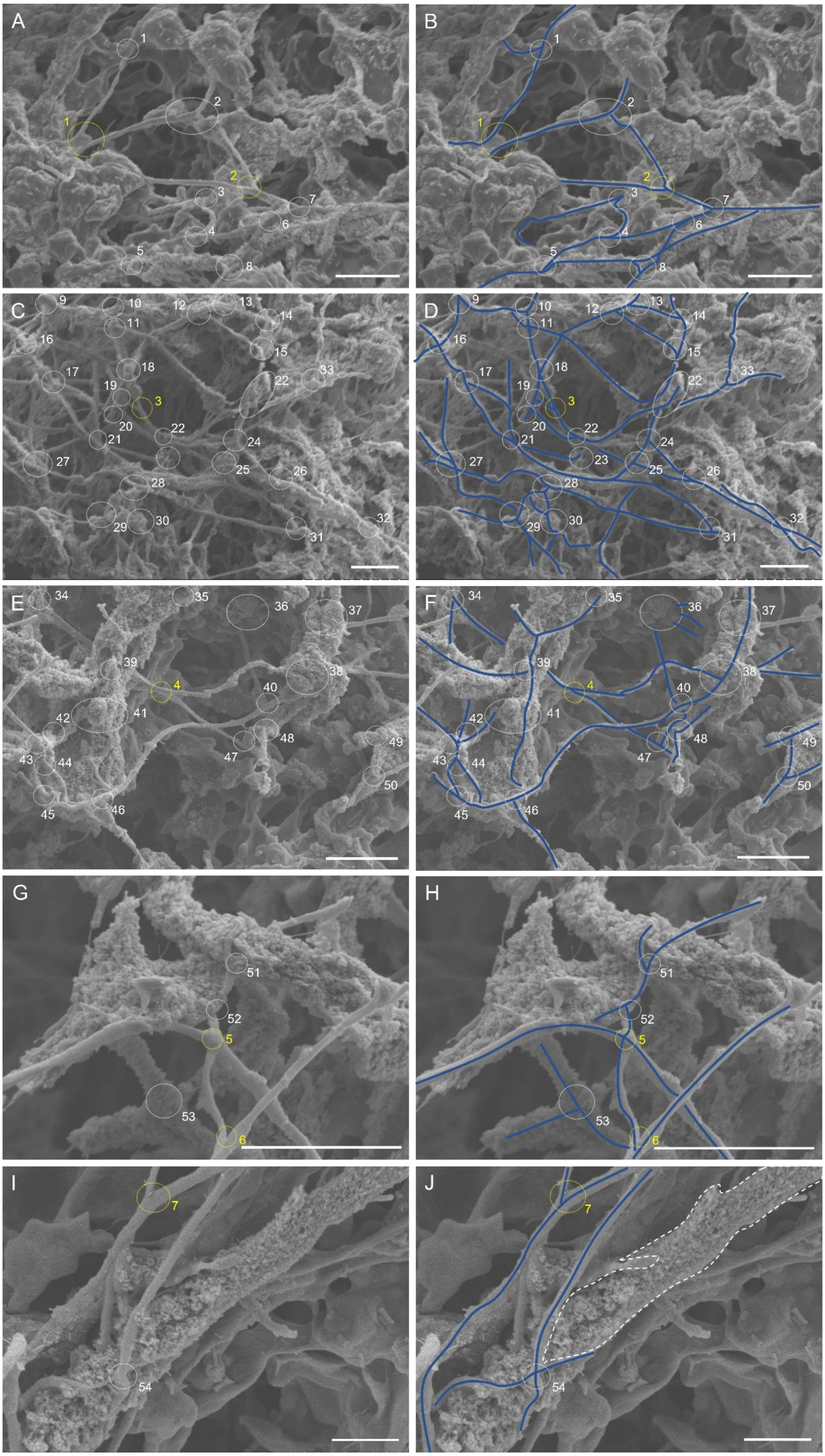
A
1 2 1 2 3 4 5 6 7 8
B
1 2 1 2 3 4 5 6 7 8
C
9 10 11 12 13 14 15 16 17 18 19 3 20 21 22 22 24 25 26 27 28 29 30 31 32 33
D
9 10 11 12 13 14 15 16 17 18 19 3 20 21 22 22 23 24 25 26 27 28 29 30 31 32 33
E
34 35 36 37 38 39 4 40 41 42 43 44 45 46 47 48 49 50
F
34 35 36 37 38 39 4 40 41 42 43 44 45 46 47 48 49 50
G
51 52 5 53 6
H
51 52 5 53 6
I
7 54
J
7 54

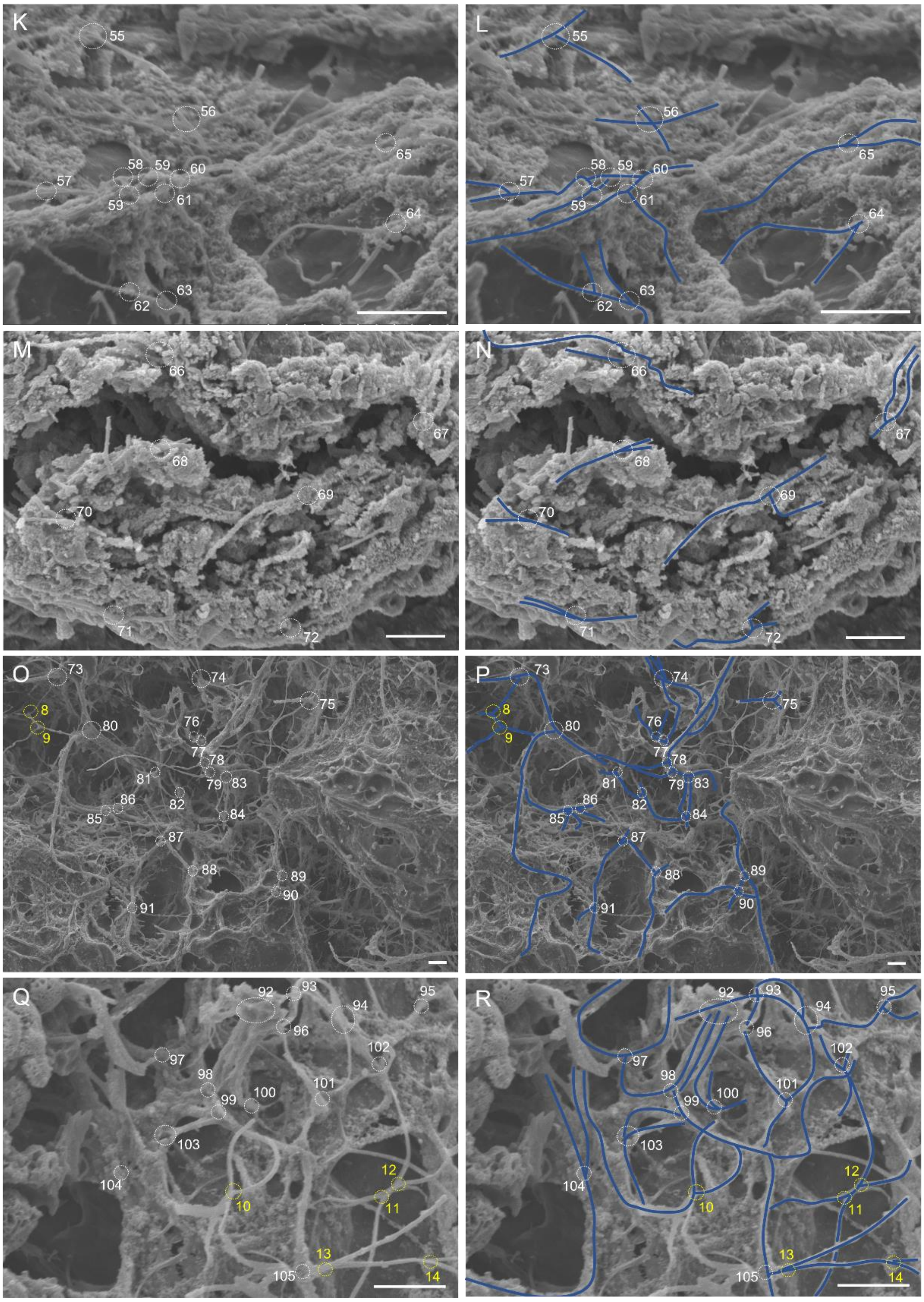

Supplementary figure 8: **Filamentous *Chloroflexi* cells form cross-linked network with junctions coated by extracellular matrix biopolymer.** Scanning electron micrographs (A-R) show filamentous *Chloroflexi* cells forming cross-linked framework with junctions (i.e., intersections formed by two or more filamentous *Chloroflexi* cells) that are non-coated and coated with extracellular matrix biopolymer. Blue solid lines in images B, D, F, H, J, L, N, P and R trace *Chloroflexi* filaments in the

biofilm. The total numbers of junctions (J_{Total} = 119) are summed from images B, D, F, H, J, L, N, P and R, depicting extracellular matrix biopolymer coated *Chloroflexi* cross-linked junctions (J_{EPS} = 105) marked with numbered white dashed circles and non-coated (J_{Free} = 14) junctions marked by numbered yellow dashed circles. The calculated number of coated filamentous junctions is likely to be underestimated as some junctions are masked by high biopolymer mass. Scale bars indicates 5µm. *Chloroflexi* junctions featured in image C exemplify *Chloroflexi*-rich regions with weaker S-layer protein interactions, indicated by thinner extracellular matrix biopolymer coating at the junctions of the filaments, compared those in image E.

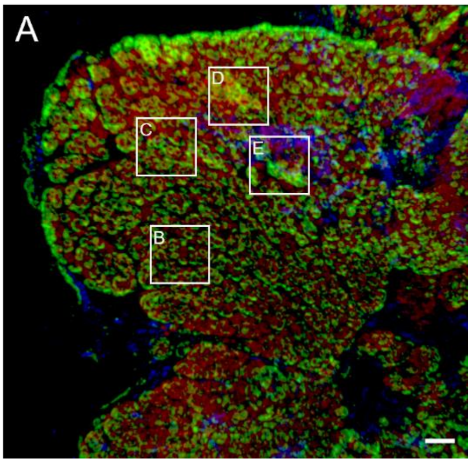

Supplementary figure 9: **Various forms of S-layer protein, BROSI-1236, on the biofilm suggests functional succession of S-layer protein from surface protein to EPS in mixed microbial community consortia.** Figure 6A with *Ca.* B. sinica FISH probe signal (Bsi630, red) showing different morphologies of S-layer protein (identified using S-layer protein antibody, green) relative to *Chloroflexi* cell (blue), suggesting the S-layer protein transitions from enveloping the cell surface to an EPS and matrix stabilising agent.

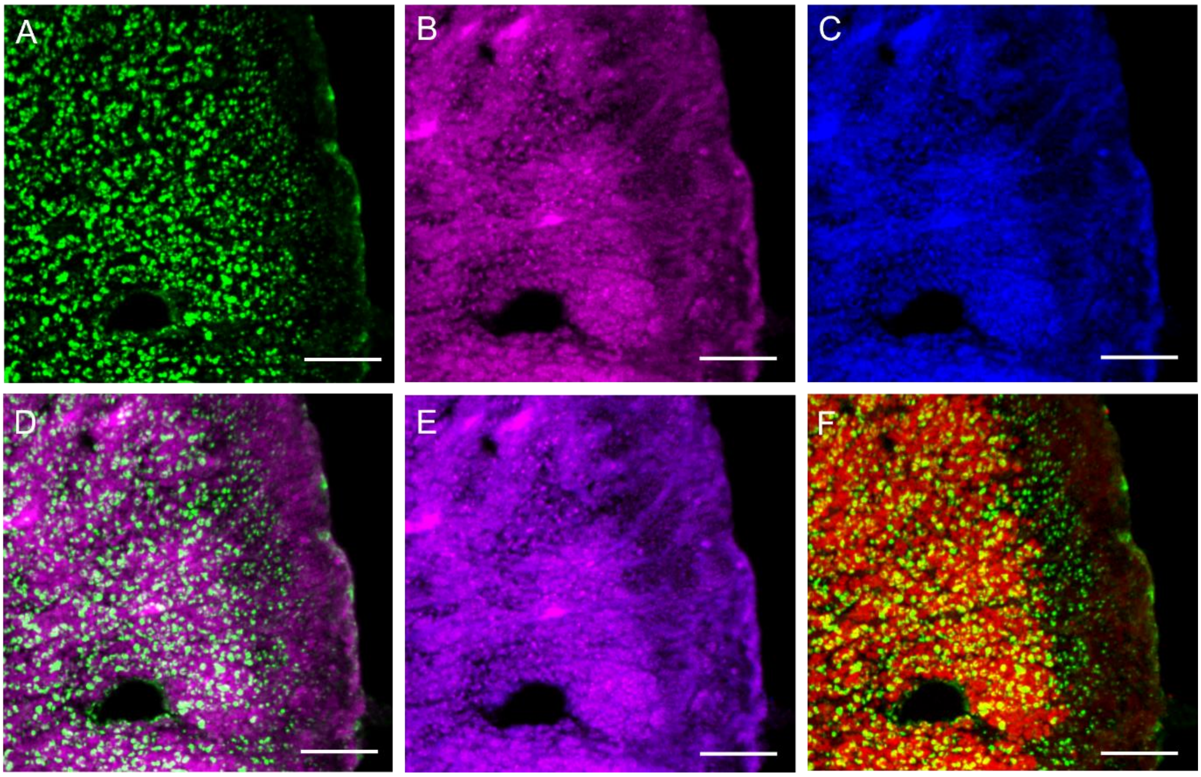

Supplementary figure 10: **S-layer protein antibody demonstrates higher selectivity on biofilm cross-section than does the general protein dye, SYPRO Ruby.** Confocal laser scanning micrograph shows anammox biofilm thin section stained with (A) S-layer protein antibody (250x dilution) visualised with Alexa Fluor 405-labelled goat anti-rabbit IgG (green), (B) general protein stain (SYPRO Ruby, magenta) and (C) general bacterial marker, EUB-338 I, II, III mix and *Chloroflexi* phylum GNSB941 FISH probes (blue). (D) Overlaying fluorescence signal of SYPRO Ruby on the S-layer antibody shows small fraction of S-layer protein staining on the biofilm cross-sections (r = 0.21; biovolume of BROSI_A1236 across four distinct thin anammox cross-section is approximately 20% of total biofilm protein). (E) Overlaying SYPRO Ruby and general bacterial marker, EUB-338 I, II, III mix and GNSB941 showed a similar staining pattern (r=0.51). (F) Overlaid image of *Ca.* B. sinica and CFX1223 *Chloroflexi* cell (stained by *Ca.* B. sinica specific Bsi630 FISH probe (red)) and S-layer protein staining. Scale bars indicate 20 µm.

$$\text{Amount of BROSI\_A1236} = \left(\frac{Biovolume\ BROSI\_A1236}{Biovolume\ SYPRO\ Ruby + Biovolume\ BROSI\_A1236}\right) * 100\%$$

Supplementary table 1. CheckM quality check of metagenome assembled genomes (MAGs) recovered within the *Chloroflexi* phylum.

Bin ID	Completeness	Contamination	Strain heterogeneity
MAGs_1 **	98.18	0.91	0.00
MAGs_2 **	77.64	1.09	0.00
MAGs_3 **	98.18	0.00	0.00
MAGs_4 **	88.18	1.01	0.00
MAGs_5 **	85.44	0.91	0.00

Footnote: High quality bins typically have < 10% contamination and > 80% completeness

Supplementary table 2. Details of oligonucleotide probes used for cryosection-FISH.

Target population(s)	FISH probe-fluorochrome	Probe sequences (5’-3’)	Reference
General bacterial	EUB338-Cy5 & EUB338-Cy3	GCT GCC TCC CGT AGG AGT	(1)
	EUB338II-Cy5 & EUB338II-Cy3	GCA GCC ACC CGT AGG TGT	(2)
	EUB338III-Cy5 & EUB338III-Cy3	GCT GCC ACC CGT AGG TGT	(2)
Ca. Brocadia sinica	Bsi630-Cy3 & Bsi630-AF488	CAT GCA GTT TCG ACC GCC AT	(3)
Chloroflexi	GNSB941-Cy5	AAA CCA CAC GCT CCG CT	(4)
	CFX1223-Cy5	CCA TTG TAG CGT GTG TGT MG	(5)

References

1. Amann RI, Binder BJ, Olson RJ, Chisholm SW, Devereux R, Stahl DA. Combination of 16S rRNA-targeted oligonucleotide probes with flow cytometry for analyzing mixed microbial populations. Appl Environ Microbiol. 1990;56(6):1919-25.

2. Daims H, Nielsen JL, Nielsen PH, Schleifer K-H, Wagner M. *In situ* characterization of Nitrospira-like nitrite-oxidizing bacteria active in wastewater treatment plants. Appl Environ Microbiol. 2001;67(11):5273-84.

3. Lu Y, Natarajan G, Nguyen TQN, Thi SS, Arumugam K, Seviour TW, et al. Species level enrichment of AnAOB and associated growth morphology under the effect of key metabolites. bioRxiv. 2020:2020.02.04.934877.

4. Björnsson L, Hugenholtz P, Tyson GW, Blackall LL. Filamentous *Chloroflexi* (green non-sulfur bacteria) are abundant in wastewater treatment processes with biological nutrient removal Microbiology. 2002;148(8):2309-18.

5. Gich F, Garcia-Gil J, Overmann J. Previously unknown and phylogenetically diverse members of the green nonsulfur bacteria are indigenous to freshwater lakes. Arch Microbiol. 2001;177(1):1-10.
